# An explorative and confirmative factor analysis of the Leadership and Management Inventory-II among staff working in elderly care

**DOI:** 10.1108/LHS-01-2023-0004

**Published:** 2023-11-14

**Authors:** Bernice Skytt, Hans Högberg, Maria Engström

**Affiliations:** Faculty of Health and Occupational Studies, University of Gävle, Gävle, Sweden and Department of Public Health and Caring Sciences, Uppsala University, Uppsala, Sweden; Faculty of Health and Occupational Studies, University of Gävle, Gävle, Sweden; Faculty of Health and Occupational Studies, University of Gävle, Gävle, Sweden; Department of Caring Sciences, Uppsala University, Uppsala, Sweden and Nursing Department, Medicine and Health College, Lishui University, Lishui, China

**Keywords:** Statistical analysis, Health leadership competencies, Leadership, Management, Nurses

## Abstract

**Purpose:**

The Purpose of the study was to investigate the construct validity and internal consistency of the LaMI among staff in the context of elderly care in Sweden.

**Design/methodology/approach:**

Questionnaire data from a longitudinal study of staff working in elderly care were used. Data were collected using the Leadership and Management Inventory. First data collection was for explorative factor analysis (*n* = 1,149), and the second collection, one year later, was for confirmatory factor analysis (*n* = 1,061).

**Findings:**

The explorative factor analysis resulted in a two-factor solution that explained 70.2% of the total variance. Different models were tested in the confirmatory factor analysis. The final model, a two-factor solution where three items were omitted, showed acceptable results.

**Originality/value:**

The instrument measures both leadership and management performance and can be used to continually measure managers’ performances as perceived by staff to identify areas for development.

## Background

Within elderly care there is a shortage of staff (Gaudenz, De Geest, Schwendimann, Zúñiga, 2019), and in addition there are reports of turnover intentions around 23% (Engström *et al.*, 2021) to 56% among staff (Gaudenz, De Geest, Schwendimann, Zúñiga, 2019). Staff-rated leadership has been linked to both staff turnover intentions (Lavoie-Tremblay *et al.*, 2022; Gaudenz, De Geest, Schwendimann, Zúñiga, 2019) and job satisfaction (Specchia *et al.*, 2021). Job satisfaction, in turn, has been found to be related to turnover intentions (Engström *et al.*, 2021; Lee, 2022) and older peoples’ satisfaction with care (Engström *et al.*, 2023). The situation within elderly care, with an increasing number of persons in need of support and care (WHO, 2016) and an aging population carrying long-term disabilities and comorbidities (Abramsson *et al.*, 2017), has for years been reported as both challenging and complex (Hagerman *et al.*, 2015). The workload for the staff is described as burdensome (Maneschiöld and Lucaci-Maneschiöld, 2021), and the levels of sick leave and turnover are reported as high (Ministry of Health and Social Affairs, 2018). Managers’ leadership and management performance have, in a systematic review, been described as important for healthy work environment (Brady Germain and Cummings, 2010). More resent, in the meta-analysis by Lake *et al.* (2019) association between work environment with job and health outcomes has been identified showing that the work environment is of importance for both patient and staff wellbeing. Thus, there is a need of high qualified leaders with good skills in both leadership and management who can support their staff, promote staff job satisfaction and prevent turnover intentions. Leaders who can manage the unit in the best way for both staff and patients. So far, there is a deficit of psychometrically sound instruments to be used in elderly care to measure management and leadership performance.

When planning for studies in elderly care, we decided to use the Leadership and Management Inventory (LaMI) (Skytt *et al.*, 2008) to learn more about first-line managers’, management–leadership performance, both self-rated and rated by their subordinates. The development of LaMI was started in 2000 (Skytt *et al.*, 2008) within health care. In the processes of finding an instrument to use when evaluating first-line nurse managers’ abilities as managers and leaders, various instruments were considered and evaluated. It was concluded that most instruments focused on leadership, and none took into consideration the health-care setting. Furthermore, the instruments that had been used to evaluate developmental activities that first-line nurse managers were involved in seemed to focus only on their role as a leader (Skytt *et al.*, 2008). As we support the posture that leadership is an important role for managers (Yukl and Gardner, 2019), it was decided to develop an instrument that evaluated both managerial and leadership skills. In 2000, the first version of the LaMI was developed based on a qualitative study. Interviews with the topic “the competent first-line nurse manager” were conducted with 19 persons working in two hospital settings within one health care region, such as hospital- nurse- and human recourses directors (six persons), department heads (five persons), registered nurses, assistant nurses, physicians (six persons) as well as one representative from the registered nurses labor union and a patients’ organization, respectively. Using an inductive approach, a pool of statements describing the performance of the first-line nurse managers were identified. Then, also based on a literature review, 56 items were formed and grouped into five topics. Presented in Skytt *et al.* ((2008) are descriptions of the construction of the first version of the inventory, which comprises 28 statements divided into three factors, and the psychometric testing used. Associations to a hospital setting were found to exist only in explicit statements regarding the knowledge of functions and decisions made in the organization.

In more recently published articles, study-specific instruments/questionnaires have been developed and used with the aim of assessing health-care managers’ competencies. Knowledge generated from these instruments is used to lay a foundation for developmental measures that can strengthen managers in their roles. The developed instruments looked at managerial and leadership competencies (Gunwan *et al.*, 2019; Isfahani *et al.*, 2015; Liou *et al.*, 2021; Lopes *et al.*, 2019; Meissner and Radford, 2015; Pillay, 2011), but the managerial levels were not clearly described, except for the instrument developed by Gunwan *et al.* (2019). The LaMI was developed to measure the performance of first-line managers. The settings where the managers were employed varied and included: primary care (Lopes *et al.*, 2019), hospitals (Gunwan *et al.*, 2019; Isfahani *et al.*, 2015; Pillay, 2011), both hospital and medical centers (Liou *et al.*, 2021) and elderly care settings (Meissner and Radford, 2015).

In the literature, there are several descriptions of different leadership styles. In studies from the nursing context, transformational leadership (Bass and Avolio, 1990), authentic leadership (Avolio and Gardner, 2005), ethical leadership (Brown *et al.*, 2005) and servant leadership (Greenleaf, 1977) are often used. Among them, transformational leadership was found to be the most studied leadership style (Hult *et al.*, 2023). These leadership styles can be described as positive, as these leaders show consideration for others. Niinihuhta and Häggman-Laitila (2022) present in their systematic review that supportive and relational leadership styles are of important for nurses wellbeing and intention to stay as well as patient outcomes. Ferreira *et al.* (2022) presented that the transformational leadership style was among nine leadership styles and the one that most positively influenced professionals, patients and institution. The transformational leadership is positive for nurses’ intention to stay (Cardiff *et al.*, 2023) and job satisfaction (Specchia *et al.*, 2021). Items in the LaMI have a resemblance to three of the four behaviors described in transformational leadership, i.e. having consideration for the individual follower’s needs and capacity, providing intellectual stimulation that, for example, supports followers’ creativity and problem-solving abilities, and instilling inspiration to followers (Bass and Avolio, 1990). Katz (1955, 1974) has suggested that managerial skills should be divided into three skill groups, an approach that is widely accepted. The human skills are related to the ability to work with people; technical skills are the knowledge of processes; and procedures and the ability to use equipment and software and conceptual skills are the ability for analytic thinking, for example, to see the organization or enterprise as a whole. These skills are also recognizable in LaMI. Thus, we thought the instrument could also be of interest in elderly care.

Accordingly, the instrument has been used in several research projects. In elderly care, e.g. a study comparing first-line managers’ self-rated leadership and management in Egypt and Sweden (Abdelrazek *et al.*, 2010) and a longitudinal one that studied relationships between first-line managers’ ratings of their empowerment as well as their subordinates’ ratings of their manager’s leadership and managerial performance. The results from the longitudinal study showed that the more access first-line managers perceived they had to structural empowerment (c.f. Kanter, 1993), the more likely it was that their staff would give themselves higher structural empowerment ratings and give higher leadership and managerial performance ratings to their managers (Hagerman *et al.*, 2017). Additionally, changes in the first-line managers’ perceptions of their access to structural empowerment are related to changes in the first-line managers’ self-rated leadership and management, which are mediated through changes in psychological empowerment (Hagerman *et al.*, 2016). When used within elderly care by both staff (Hagerman *et al.*, 2017) and first-line managers (Hagerman *et al.*, 2016; Abdelrazek *et al*, 2010), the instrument shows good internal consistency (Hagerman *et al.*, 2016; Abdelrazek *et al.*, 2010) and few missing data but has never tested for construct validity within elderly care. To build strong research within the area, there is a need of psychometrically tested instruments within elderly care.

The LaMI has been used for more than 10 years in hospitals as well as in elderly care settings. Since our experiences using the LaMI have been satisfactory and we have not found any other health care and context-specific instrument developed and/or used in elderly care, we found it important to investigate the construct validity of the LaMI when used in elderly care.

## Methods

### Aim

The aim of the study was to investigate the construct validity and internal consistency of the LaMI among staff in the context of elderly care in Sweden.

### Design

This study is part of a larger longitudinal study with data collected on two occasions, one year apart. Responses from the first data collection (T1) have been used for the explorative factor analysis (EFA), and data from the second (T2) have been used for the confirmatory factor analysis (CFA) (Hagerman *et al.*, 2017).

### Data collection

A coded questionnaire, along with written information about the study and a stamped return envelope, was sent to the participants at their workplaces. Two reminders were sent to nonresponders (for a detailed description, see Hagerman *et al.*, 2017).

### Sample

The study population consisted of nurse aides, licensed vocational nurses, registered nurses, physiotherapists and occupational therapists working in elderly care (nursing homes or home-help services) in five municipalities in Sweden.

The number of participants in the two samples was 1,149 (T1) and 1,061 (T2), with a mean age of 47.0 (T1) and 47.6 (T2) (see Table 1). The majority of the participants in both samples had worked with the first-line manager only when they had the role of manager. About 78–79% of the participants in the two samples indicated that they were rather well to very well acquainted with their first-line manager.

### Instrument

The LaMI includes, after the described psychometric testing (Skytt *et al.*, 2008), 28 items distributed into three factors:

1. interpersonal skills and group management;
2. achievement orientation; and
3. overall view of the organization and political savvy.

The responses, with high scores being more desirable, are made on a five-point scale where 1 = not at all, 2 = to a small extent, 3 = to some extent, 4 = to a large extent and 5 = to a very large extent. Factor scores and total score items can be transformed, i.e. summed and divided by the highest possible score in that factor/total scale, and thereafter multiplied by 100. An alternative is to calculate factor or total scores by summing the scores and dividing them by the number of items. Higher scores indicate better leadership and management performance.

The LaMI can be used in three ways:

1. self-assessment by first-line managers;
2. assessment of first-line managers by their subordinates; and
3. assessment of first-line managers by their superiors.

The wording in the instrument has been adapted to suit the context of elderly care. An example of the difference in the wording from the hospital vs the elderly care version is: knows about and understands how the hospital functions at the organizational level/knows about and understands how the organization functions at the different levels.

### Data analysis

Data were analyzed using IBM SPSS Version 22 (SPSS Inc., Chicago, IL, USA). An EFA using principal component analysis (PCA) with varimax rotation was performed on data from the T1 participants with no missing values. Scree plot and eigenvalues were used to determine the number of factors. To test the theoretical and empirical models from the EFA, we used confirmative factor analysis (CFA). The CFA was made using IBM AMOS and data from the T2 participants with no missing values. Two estimation methods were used: the maximum likelihood (ML) method and the Bayesian method. The specification included means to set the scales of the unobserved factors and residuals. We fixed the variances of the unobserved factors and the residuals to one. To determine the model’s goodness-of-fit, we used the following fit indices: model Chi^2^; relative Chi^2^ (Chi^2^/df), where df is degrees of freedom; comparative fit index (CFI); and root mean square error of approximation (RMSEA) with a *p*-value (PCLOSE). These fit indices were used to indicate whether the specified structure may be confirmed and are in line with the recommendations in Hooper *et al.* ((2008). Model Chi^2^ and RMSEA are examples of absolute indices, i.e. indices of how well the model covariances match the observed covariances (Byrne, 2010; Hooper *et al.*, 2008; Kääriäinen *et al.*, 2011). CFI is an example of relative fit index, which compares the specified model to a null model in which all variables are uncorrelated (Byrne, 2010; Hooper *et al.*, 2008; Kääriäinen *et al.*, 2011). Furthermore, to get some idea of which re-specification would result in the greatest improvement in model fit, we used modification indices (MI) and standardized residuals (SR) (Byrne, 2010). There are an extensive number of recommendations regarding which indices to use and the range of values of the indices required to indicate good or acceptable model fit. The levels of the diagnostic fit indices that we have chosen are: CFI > 0.90 to indicate a good fit and preferable, >0.95 to be good; and RMSEA < 0.05–0.06 to be good, but some recommend < 0.08 to be acceptable (Byrne, 2010; Hooper *et al.*, 2008; Hu and Bentler, 1999). The p-values (PCLOSE) for the testing of the null hypothesis of RMSEA ≤ 0.05 should be *p* > 0.05. Moreover, we also regarded values of MI over 100 and SR over 7 to suggest which parameters to re-specify. We did not rely entirely on MI or SR to decide on re-specification. Sound theoretical arguments were considered more important.

To evaluate the robustness of the ML estimates to ordinal-scaled categorical observed variables, we also estimated the factor loadings using Bayesian estimation. This is the methodological approach to the analysis of categorical variables in AMOS (Byrne, 2010). Bayesian estimation does not change the goodness-of-fit measures but leads to different estimates of factor loadings and covariances. By comparing these estimates and evaluating estimation diagnostics, we were able to assess the consequences of ordinal scaled variables for CFA.

Internal consistency was calculated using Cronbach’s alpha (α). Descriptive statistics were used to describe sample characteristics as well as total and factor scores.

### Ethical considerations

The Regional Ethical Review Board approved the study in 2010 (Reg.no. 2010/192). Confidentiality was guaranteed, and participation that was strictly voluntary could be discontinued at any time without explanation.

## Results

### Explorative factor analysis

A PCA with varimax rotation was used to determine construct validity for the LaMI using sample T1 participants with no missing data (*n* = 1,149). Bartlett’s test of sphericity was significant (*p*≤ 0.001), and Kaiser–Meyer–Olkin’s measure of sampling adequacy was 0.978, which justified our proceeding with the factor analysis, i.e. a solution where two factors are able to explain 70.2% of the total variance. The factors with their corresponding items were interpreted based on their content, the initial development and earlier psychometric tests of the LaMI. One of the factors was interpreted as corresponding to the role/assignment/task as a manager and is hereafter labeled “management” (factor loadings ranging from 0.541 to 0.867, 10 items). The other factor was interpreted as corresponding to “leadership” (factor loadings ranging from 0.612 to 0.858, 18 items), see Table 2. Cross-loading was found for item number 6, and after an inspection of content, this item was ascribed as a management factor. There was no cross-loading for the other items. These results reveal that, in general, these two factors correspond to the development of the LaMI and the grouping of the initial statements as well as to previous results of the construct validity of the LaMI for second-order factor analysis. For item level, the mean ranged from 3.2 (deals with problems and conflicts) to 4.2 (knows about financial conditions), the median ranged from 3 to 4, and 11 items had a value of 5 for quartile 3. The possible range for the items is 1–5.

### Confirmatory factor analysis

Our basic model in the CFA was the two-factor model with all 28 items included. Items 1–10 were specified to load on factor 1, and items 11–28 to load on factor 2. The factors were specified not to correlate in accordance with the PCA, in which the basic factors were rotated by the varimax orthogonal rotation. The goodness-of-fit indices for the basic model were not satisfactory, see Table 3.

**Figure 1.**
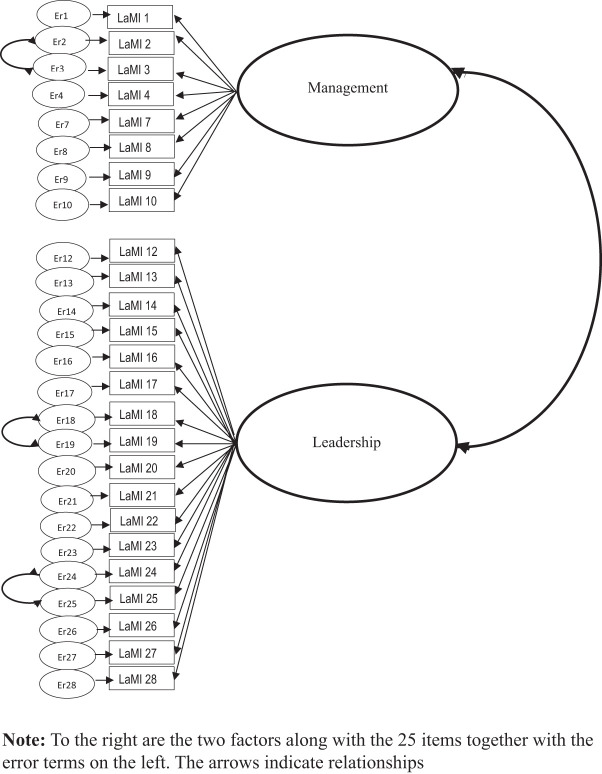
Confirmatory factor model for the final two-factor solution LaMI-II

One obvious re-specification was to allow the two factors to correlate. To get further ideas on how to modify the specification, we looked at MI and SR, which led us to look further at items 2, 5, 6 and 11 and to consider some correlated error terms. This was also in complete agreement with theoretical considerations. All modified models included correlated factors. Moreover, the first modified model showed better but not quite satisfactory goodness-of-fit indices. In the second and final modified model, items 5, 6 and 11 were excluded, and correlation between error terms for items 18 and 19 and items 24 and 25 were allowed. The models differ in regard to whether item 2 was included or not and whether error terms for items 2 and 3 were allowed to correlate. The final model, hereafter labeled LaMI-II, is described in Figure 1 and Table 4. Goodness-of-fit indices are shown in Table 3, and the instrument can be found in an appendix. The factor loadings were in agreement with factor loadings from the EFA, and we found them to be of acceptable size. All factor loadings were statistically significant. The factor loadings estimated by the ML method and the Bayesian method are shown in Table 5.

### Internal consistency

Cronbach’s alpha for the two factors in the final solution were 0.951 and 0.972, respectively (see Table 4). For the LaMI-II total, the α-value was 0.971.

## Discussion

The instrument showed acceptable construct validity and can be recommended for use in elderly care for staff ratings of their managers’ performance. Our results pertaining to elderly care show a two-factor structure for the LaMI-II with 25 items. This differs from the original hospital sample version, which resulted in 28 items divided into three factors. The second-order analysis of the hospital sample (Skytt *et al.*, 2008) is, however, largely consistent with the results from the present study. The two-factor solution in the EFA explained 70% of the total variance, with factor loading varying from 0.543 to 0.863, whereas the earlier three-factor solution from two different samples (Skytt *et al.*, 2008) explained 64% and 66% of the total variance, respectively, and had factor loadings varying from 0.427 to 0.816. Comrey and Lee (1992) suggest that factor loadings >0.55 are considered good and loadings >0.45 are fair. Thus, all except one with a factor loading of 0.543 could be considered as good for the EFA two-factor solution (CFA all >0.74). On the other hand, the EFA three-factor solution (Skytt *et al.*, 2008) for the two included samples had two, respectively, three items with factor loadings below 0.55.

When comparing our results with other instruments, the number of items and factors differ. The competencies asked for are described in more detail in some instruments than in others. Four factors and 23 items (Liou *et al.*, 2021) and six factors with 46 items are described in Lopes *et al.* ((2019). Three studies presented seven factors, with 24 items in Isfahani *et al.* (2015), 43 items in Gunwan *et al.* ((2019) and 51 items in Pillay (2011). Meissner and Radford (2015), on the other hand, presented 17 items and no factors. Construct validity has been tested by performing a PCA (Liou *et al.*, 2021; Lopes *et al.*, 2019) and a CFA in four of the studies (Gunwan *et al.*, 2019; Liou *et al.*, 2021; Lopes *et al.*, 2019; Pillay, 2011). The LaMI-II now consists of 25 items and could be judged as feasible to use in surveys together with instruments measuring other variables, which is often the case in research and for organizations’ human resource surveys. Internal consistency for factors and total score for the LaMI-II are good but could also be considered as quite high. This indicates that the number of items used to measure the concept is redundant. Cronbach’s alpha values between 0.70 and 0.95 have been recommended (Terwee *et al.*, 2007). Three of the studies have presented Cronbach’s alpha values. The study by Gunwan *et al.* ((2019) reported values between 0.71 and 0.90; Liou *et al.* (2021) reported 0.74–0.90; and Pillay (2011) reported 0.81–0.93, i.e. all within recommended values (Terwee *et al.*, 2007). For some items, Q3 reached the highest possible score, which might indicate a ceiling effect (two of these were deleted in LaMI-II). Ceiling effect, in turn, could be problematic when measuring responsiveness to the detection of important changes/improvements over time (Terwee *et al.*, 2007). However, the factor level of the LaMI has previously been shown to measure improvements over time when used in experimental design (Skytt *et al.*, 2011).

In the LaMI-II, the following three items have been omitted: contributes to goal formulations, focuses on goal achievement and presents understandable information. The managerial skills focusing on the goals of the organization can still be found in the present solution in items dealing with the ability to see the whole picture, unit’s goals in connection with the organizations, and being clear about requirements and expectations. Skills focusing on being able to present understandable information can also still be found in the LaMI-II in items dealing with the manager’s ability to describe and explain decisions (two items) and being clear about requirements and expectations (one item).

The CFA also showed that six of the items correlated, and the correlations are noted within the respective factors. Two items correlated in the factor management and four in the factor leadership. Based on the CFA and theoretical reasoning in the research group, we allowed these items (error terms) to correlate. In the factor management, there is a correlation between item 2 that deals with their ability to describe and explain decisions and item 3 that deals with their ability to describe and explain the consequences of decisions. It seems reasonable that those two items correlate since the consequences of a decision logically follow the description and explanation of the decision made in the organization. In the factor leadership, some of the items have their focus on the operations of the unit, which can be understood as the goals of the service even though the word goal is not explicitly mentioned. Items 18 and 19 were allowed to correlate as well as items 24 and 25 (Table 4 and Figure 1). The items provide clarity regarding requirements and expectations (18), making sure that what has been agreed upon gets done (19), having an inspiring and motivating approach (inspires coworkers item 24) and promoting and encouraging cooperation (improves team spirit item 25).

We would like to underscore that the factor leadership in the LaMI-II addresses the formal leadership, skills and competencies that characterize a competent first-line manager. The items in our solution also reflect Katz’s human, conceptual and technical three-skill approach. In the LaMI-II’s factor leadership, “human skills” are dominant. Human skills involve being able to work with others, and that could be with subordinates in the manager’s own unit as well as persons in other units. Some examples are to accept others’ opinions and points of view even when they differ from their own, to see the needs of others and to have the ability to motivate others. Items that address “technical skills”, i.e. knowledge and abilities that are specific to the job, are also found in the leadership factor. Items that related to “technical skills” are e.g. to take the initiative to work on improvements and to delegate tasks correctly. In the LaMI-II’s factor management, conceptual skills are described, i.e. to see the organization as a whole, and understand how decisions and conflicting situations can affect the operations. Conceptual skills have been described as being of special importance in higher levels of management. In our material, conceptual skills can be exemplified as being able to understand the organization as well as describe and explain consequences of decisions that are made in the organization. Additionally, “technical skills” can be found in the managerial factor since these skills address knowledge of administrative facts that are included in the first-line manager’s scope of responsibility (Katz, 1955, 1974).

In a study by McCarthy and Fitzpatrik (2009), a competency framework for nurse managers in Ireland is described. Besides generic competencies, they present competencies identified for front line-, middle- and director levels. When making comparisons with our results, it can be seen that skills in our instrument can be found at all three managerial levels in that framework. It is interesting to compare our instrument with the one presented by Gunwan *et al.* ((2019), as they have also focused on first-line managers’ competencies. The competencies “facilitating spiritual nursing care”, “self-management” and “utilizing informatics (in nursing practice)” are not included in LaMI-II. The other competencies described can be found in our instrument, even though there are less items in LaMI-II and the wording is less specific.

In our studies in elderly care focusing on the work life of personnel and their managers LaMI has given us information about one aspect of importance, the first-line managers’ management–leadership performance (Hagerman *et al.*, 2016, 2017). Vidman and Strömberg (2020) interviewed personal in elderly care to learn more about what can contribute to healthy work environment. Their result shows that the themes *What to do, How to do it* and *Who to be* simultaneously form the opinion on the leadership that contributes to a healthy work environment. The subtheme maintaining order (in *What to do)* shows resemblances with the factor management in LaMI-II. The items in factor leadership in LaMI-II can be recognized in problem-solving (in *What to do*), trusting the staff and Interaction between leaders and staff (in *How to do it)* and leader’s attributes (in *Who to be)* in the description by Vidman and Strömberg (2020). That supports our decision that LaMI-II is relevant to use when studying management–leadership performance.

Expectations of nurse managers’ competences will differ depending on the culture and country’s requirements and attitudes, the context and the organizational level. That expectations can differ over time should also be taken into consideration.

### Method discussion

The strengths of the study are that both EFA and CFA, in two different data sets, are used to test the construct validity of the instrument. Estimation methods such as the Maximum Likelihood method could be questioned when used for ordinal data. The model was also tested using the Bayesian method, and only minor deviations between these two could be noted (see Table 5). A shorter version of the instrument that captures the concept and still retains good internal factor consistency might make the instrument easier and less tiring to use, especially when several instruments are used together in a study. Limitations are that LaMI has so far mostly been used in Sweden and in elderly care, except for a few studies in health care. To develop and validate an instrument, testing is needed in different contexts, such as hospital and nonhospital settings, different cultures and countries, and with different methods. Concurrent validity as well as testing and re-testing are needed in future studies.

## Conclusions

The situation within elderly care is challenging and complex. Staffs’ workload is heavy; there is a shortage of staff; sick leave and turn over intention are high. In this situation, there is a need for qualified first line managers. In this study, we have investigated the construct validity and internal consistency of an instrument addressing first-line managers leadership and management abilities in elderly care. The leadership and management inventory (LaMI II) consists of two factors: leadership and management and includes 25 items. LaMI II showed acceptable psychometric results and can be recommended for use. If LaMI II is used by organizations for human resource surveys with subsequent development activities based on the survey results, it can hopefully help to improve the work situation for staff in elderly care, improve job satisfaction and decrease job turnover. However, if used, it needs to be complemented with instrument that capture the structural conditions for the manager, as structural conditions in turn are related to managers’ leadership and management performance.

## Implications for management in health services

This instrument, the LaMI-II, can be used when studying relationships between first-line managers and their subordinate’s ratings of the first-line manager’s performance. It can also be used to measure the first-line managers’ performance in relation to work-related measures such as psychological and structural empowerment. The LaMI-II can be used by organizations for human resource surveys. If the results indicate a need for development activities, the LaMI has been found in previous research to be sensitive to changes over time, and there is congruence between the focus of the development activities and the various skills (Skytt *et al.*, 2011).

## Figures and Tables

**Table 1.** Sample characteristics

	Sample T1 *n* = 1,149		Sample T2 *n* = 1,061	
Characteristics	*Mean (SD)*	*Min/Max*	*Mean (SD)*	*Min/Max*
Age of the participants (years) (T1 *n* = 970) (T2 *n* = 1,053)	47.0 (10.3)	19/67	47.6 (10.1)	22/66
	*Frequency*	*Percent*	*Frequency*	*Percent*
Gender women/men	1,107/42	96.3/3.7	1,030/31	97.1/2.9
Acquaintance with the FLM				
-Almost not at all	241	21.0	209	19.7
-Rather well	532	46.3	486	45.8
-Well	278	24.2	276	26.0
-Very well	88	7.7	82	7.7
-Missing	10	0.9	8	0.8
Have worked together with the person before they became manager				
-Yes	105	9.1	115	10.8
-No	1,020	88.8	930	87.7
-Missing	24	2.1	16	1.5

Notes: T1 = Time 1; T2 = Time 2; SD = Standard Deviation; FLM = First-Line Manager

**Source:** Authors’ own work

**Table 2.** Principal component analysis with varimax rotation for sample T1 (*n* = 1,149), rotated factor loadings for the leadership and management inventory (LaMI)

Item in short form/factor name	Leadership	Management	Mean (SD)	Md(Q_1_–Q_3_)
1 Knows about the functions of the organization		*0.824*	4.0(0.9)	4(4–5)
2 Describes and explains decisions		*0.867*	3.8(1.0)	4(3–4.5)
3 Can describe and explain the consequences of decisions		*0.842*	3.6(1.1)	4(3–4)
4 Sees the whole picture, unit’s goals in connection to organization’s	0.373	*0.776*	3.8(0.9)	4(3–4)
5 Contributes to the formation of a goal	0.487	*0.657*	3.9(0.9)	4(3–5)
6 Focus on goal achievement	0.578	0.541	3.8(1.0)	4(3–5)
7 Knows where to find help	0.346	*0.786*	3.9(1.0)	4(3–5)
8 Knows how to make an impact		*0.823*	3.7(1.0)	4(3–4)
9 Knows about financial conditions		*0.814*	4.2(0.9)	4(4–5)
10 Knows about laws, agreements and guidelines	0.361	*0.695*	3.8(1.0)	4(3–5)
11 Presents understandable information	*0.612*	0.558	3.8(1.0)	4(3–5)
12 Listens	*0.811*		3.6(1.1)	4(3–4)
13 Interacts easily	*0.736*	0.317	3.9(0.9)	4(3–5)
14 Gives constructive criticism	*0.780*		3.6(1.0)	4(3–4)
15 Is tolerant of people’s mistakes	*0.673*		3.6(1.0)	4(3–4)
16 Consistent - what is said is done	*0.798*		3.5(1.1)	4(3–4)
17 Responsible for own mistakes	*0.821*		3.6(1.1)	4(3–4)
18 Clear about requirements and expectations	*0.683*	0.372	3.8(1.0)	4(3–5)
19 Makes sure that things get done	*0.763*	0.312	3.7(1.0)	4(3–4)
20 Values the efforts of all personnel	*0.810*		3.6(1.1)	4(3–5)
21 Active in personnel development	*0.838*	0.320	3.6(1.1)	4(3–4)
22 Initiates improvements	*0.763*	0.360	3.7(1.0)	4(3–4)
23 Trusts co-worker’s competence	*0.760*	0.339	3.9(1.0)	4(3–5)
24 Inspires coworkers	*0.836*		3.5(1.1)	4(3–4)
25 Improves team spirit	*0.858*		3.4(1.1)	4(3–4)
26 Delegates correctly	*0.780*		3.6(1.0)	4(3–4)
27 Encourages cooperation	*0.747*		3.5(1.0)	4(3–4)
28 Deals with problems and conflicts	*0.839*		3.2(1.2)	3(2–4)
Number of items	18	10	28	28

Notes: *Italics face text* for loadings indicates the respective factors when highest in that factor. Values below 0.300 are not showed in the table

**Source:** Authors’ own work

**Table 3.** Goodness-of-fit indices for the alternative models in the confirmatory factor analysis of the leadership and management Inventory-II (LaMI-II)

Model	Chi^2^	Chi^2^/df	CFI	RMSEA	PCLOSE	High MI†	High SR††
*Basic model*	3,577	10.248	0.894	0.093	< 0.0001	5	3
1^st^ modified	2,049	8.162	0.929	0.082	< 0.0001	2	0
2^nd^ modified	1,832	7.356	0.937	0.077	< 0.0001	0	0
3^rd^ final model	1,933	7.133	0.938	0.076	< 0.0001	0	0

Notes: †Modification index >100 for relevant parameters; ††standardized residual covariances >7

Abbreviations: CFI: comparative fit index, RMSEA: root mean square error of approximation, MI: modification index, SR: standardized residuals

**Source:** Authors’ own work

**Table 4.** Final solution for LaMI-II, factor names, items, Cronbach’s alpha (α), means, median and measures of dispersion (at time 2)

Mean and medium	Management	Leadership	LaMI II Total
	1 Knows about the functions of the organization	11 Presents understandable information	
	2 Describes and explains decisions	12 Listens	
	3 Can describe and explain the consequences of decisions	13 Interacts easily	
	4 Sees the whole picture, unit’s goals in connection to the organization’s	14 Gives constructive criticism	
	5 Contributes to goal formulation	15 Is tolerant of people’s mistakes	
	6 Focus on goal achievement	16 Consistent - what is said is done	
	7 Knows where to find help	17 Responsible for own mistakes	
	8 Knows how to make an impact	18 Clear about requirements and expectations	
	9 Knows about financial conditions	19 Makes sure that things get done	
	10 Knows about laws, agreements and guidelines	20 Values the efforts of all personnel	
		21 Active in personnel development	
		22 Initiates improvements	
		23 Trusts co-worker’s competence	
		24 Inspires coworkers	
		25 Improves team spirit	
		26 Delegates correctly	
		27 Encourages cooperation	
		28 Deals with problems and conflicts	
α	0.95	0.97	0.97
Mean (SD)	4.0 (0.8)	3.7 (0.8)	3.8 (0.8)
Md (Q_1_–Q_3)_	4.0 (3.5–4.6)	3.8 (3.3–4.3	3.8 (3.4–4.3)
	*Transformed values*		
Mean (SD)	79.1 (16.0)	74.3 (16.7)	75.8 (15.1)
Md (Q_1_–Q_3)_	80.0 (70.0–92.5)	75.5 (65.9–85.9)	76.8 (67.2–86.4)

Notes: Crossed out text illustrates that the item is omitted;

α = Cronbach’s alpha; SD = standard deviation; Md = median; Q = quartiles

**Source:** Authors’ own work

**Table 5.** Estimates of factor loadings from CFA models. The final model using maximum likelihood method, bayes final is the final model estimated by Bayesian method

Factor loadings	Final model	Bayes final*
*Management*		
Item 1	0.679	0.685
Item 2	0.835	0.842
Item 3	0.832	0.839
Item 4	0.772	0.779
Item 7	0.775	0.781
Item 8	0.830	0.838
Item 9	0.700	0.707
Item 10	0.796	0.802
*Leadership*		
Item 12	0.828	0.838
Item 13	0.698	0.706
Item 14	0.845	0.854
Item 15	0.715	0.722
Item 16	0.861	0.871
Item 17	0.845	0.853
Item 18	0.701	0.709
Item 19	0.777	0.784
Item 20	0.871	0.879
Item 21	0.908	0.917
Item 22	0.843	0.852
Item 23	0.790	0.799
Item 24	0.915	0.924
Item 25	0.929	0.938
Item 26	0.770	0.778
Item 27	0.780	0.789
Item 28	0.917	0.927

Notes: *Convergence was achieved after 500 so-called burn-in samples and further 2*89,500 random draws. CFA: confirmatory factor analysis

**Source:** Authors’ own work
